# A Novel Synthesis of ZnO Nanoflower Arrays Using a Lift-Off Technique with Different Thicknesses of Al Sacrificial Layers on a Patterned Sapphire Substrate

**DOI:** 10.3390/nano12040612

**Published:** 2022-02-11

**Authors:** Hsien-Wei Tseng, Ching-Shan Wang, Fang-Hsing Wang, Han-Wen Liu, Cheng-Fu Yang

**Affiliations:** 1 College of Artificial Intelligence, Yango University, Mawei District, Fuzhou 350015, China; hsienwei.tseng@gmail.com; 2 Graduate Institute of Optoelectronic Engineering, National Chung Hsing University, Taichung 402, Taiwan; ching.shan73@msa.hinet.net (C.-S.W.); hwliu@dragon.nchu.edu.tw (H.-W.L.); 3 Department of Chemical and Materials Engineering, National University of Kaohsiung, Kaohsiung 811, Taiwan; 4 Department of Aeronautical Engineering, Chaoyang University of Technology, Taichung 413, Taiwan

**Keywords:** lift-off technique, patterned sapphire substrate, aluminum sacrificial layer, ZnO nanoflower arrays

## Abstract

A novel method to synthesize large-scale ZnO nanoflower arrays using a protrusion patterned ZnO seed layer was investigated. Different thicknesses of aluminum (Al) film were deposited on the concave patterned sapphire substrate as a sacrificial layer. ZnO gel was layered onto the Al film as a seed layer and OE-6370HF AB optical glue was used as the adhesive material. A lift-off technique was used to transfer the protrusion patterned ZnO/AB glue seed layer to a P-type Si <100> wafer. The hydrothermal method using Zn(CH_3_COO)_2_ and C_6_H_12_N_4_ solutions as liquid precursors was used to synthesize ZnO nanoflower arrays on the patterned seed layer. X-ray diffraction spectra, field-effect scanning electron microscopy, focused ion beam milling (for obtaining cross-sectional views), and photoluminescence (PL) spectrometry were used to analyze the effects that different synthesis times and different thicknesses of Al sacrificial layer had on the properties of ZnO nanoflower arrays. These effects included an increased diameter, and a decreased height, density (i.e., number of nanorods in μm^−2^), total surface area, total volume, and maximum emission intensity of PL spectrum. We showed that when the synthesis time and the thickness of the Al sacrificial layer were increased, the emission intensities of the ultraviolet light and visible light had different variations.

## 1. Introduction

Zinc oxide (ZnO) is used in a variety of materials with many different applications, including nano-ZnO/Ag in antimicrobial applications [1], and Co-doped ZnO sensors, especially CoZnO-3, exhibited a superior performance in sensing triethylamine (TEA) [2]. ZnO-based materials are versatile because they have many different properties [3], including ultraviolet (UV) and visible light luminescence [4], high electrical conductivity [5], and high piezoelectricity [6]. ZnO can be easily synthesized into nanostructures at low temperatures using a solution process, because this growing process can synthesize the ZnO nanostructure materials at temperatures lower than 100 °C [5,7,8]. Therefore, this synthesis technology can be easily used to fabricate ZnO-based nanomaterials. These ZnO nanostructures include thin film, nanoparticles, hollow spheres, nanorods, and nanoflowers, which all have good chemical stability, are nontoxic, and can be synthesized at a low cost. Therefore, ZnO-based nanostructured materials have become one of the most common metal oxide photocatalysts [7,8]. The properties of ZnO-based materials are mainly influenced by their fabrication method, structure, and outward appearance, including surface defects and optical bandgap characteristics. Methods to increase the pore structure and, thus, the surface area for optimal dye regeneration and sensitization have been investigated in ZnO-based materials with three-dimensional (3D) structures and morphologies [9]. The surface area of a ZnO-based nanomaterial is an important factor that influences its optical properties.

Nanomaterials with high surface areas have a better ability to absorb scattered light or to emit excited light; thus, the light harvesting efficiency or light emission efficiency of these materials are improved. There is interest in increasing the surface area of ZnO nanomaterials with one-dimensional (1D) morphologies, such as ZnO nanorods and ZnO nanowires, which would be advantageous in terms of light harvesting and emission [10], which can be used to enhance the efficiency of the fabricated devices [11]. There have been many efforts to combine the 3D synthesis model into 1D ZnO nanostructures to increase their effective surface areas. For example, researchers have tried to synthesize nanorods that grow vertically from the substrate into randomly branched nanoflower structures. Efforts have been made to optimize the fabrication technology of ZnO nanoflowers with controllable and repeatable morphologies to form ZnO nanoflower arrays. For example, Yu et al. synthesized ZnO nanoflower arrays on a patterned sapphire substrate with a periodical structure using a two-step method [12]. Guo et al. combined laser direct writing with the hydrothermal method to synthesize ZnO on polymethyl methacrylate [13]. Kim et al. synthesized double-faced ZnO nanoflowers by first transferring ZnO-coated microparticles onto a polyimide substrate that had been coated with polyvinyl phenol and then removing the microparticles [14]. Bourfaa et al. arranged different surface positions of the seed layer to control the synthesis of ZnO nanorods and ZnO nanoflowers [15].

Although there are many methods that have been used to synthesize 1D ZnO nanostructured materials, the hydrothermal method is the most commonly used technology. The hydrothermal method is a simple, useful, and economical way to synthesize 1D ZnO nanostructured materials at low temperatures [16]. ZnO nanoflowers have different outstanding applications in diverse fields beyond themselves. For example, they can be used in water detoxification and environmental remediation [17]. In addition, ZnO nanoflowers have a detoxification ability against the methylene blue, rhodamine 6G, and oxytetracycline molecules solution in water [18]. Therefore, the hydrothermal method was used in this research to synthesize ZnO nanoflowers on a prepared protrusion patterned ZnO seed layer. In this study, we investigated the synthesis of ZnO nanoflower arrays using a lift-off technique with different thicknesses of Al sacrificial layers on a patterned sapphire substrate. A ZnO film/OE-6370HF AB optical glue multilayer film coated with a patterned concave sapphire substrate was moved to a P-type silicon (Si) <100> wafer to form a protrusion patterned ZnO seed layer. The hydrothermal method was used to synthesize ZnO nanorods using 0.02 M C_6_H_12_N_4_ (hexamethylenetetramine (HMT)) and Zn(CH_3_COO)_2_ (zinc acetate) solutions as precursors. The synthesized ZnO nanorods grew perpendicular to the protrusions in the ZnO seed layer in a radial direction, such that they formed a hierarchical structure. Therefore, this method was successfully used to synthesize ZnO nanoflowers.

The important novel technology presented in this study was that of a protrusion patterned ZnO seed layer used to fabricate ZnO nanoflowers that possessed a remarkable repeatability to form ZnO nanoflower arrays. In the past, Guo et al. used the spinning method to layer PMMA 1 μm thick onto a GaN/LiAlO_2_ substrate; then they used a laser to write the holes in the matrix. Then they used the hydrothermal method to synthesize the nanoflowers on the prepared substrate [13]. A shortcoming of this research is that they used the expensive GaN/LiAlO_2_ substrate and a laser to prepare that substrate. Katiyar et al. used the ultrasonic-assisted hydrothermal method to grow the ZnO nanoflowers at a low temperature with no substrate [19]. A shortcoming of this research is that they could not synthesize the ZnO nanoflowers in the array or matrix structure. Huo et al. applied the self-assembled method to a monolayer polystyrene (PS) sphere, and then they used the matrix PS spheres to synthesize the ZnO nanorods with a matrix structure [20]. However, Huo et al. could not synthesize the ZnO nanorods in the structure of the ZnO nanoflowers. In order to improve on these shortcomings, we investigated a novel and easy method to synthesize the ZnO nanoflower arrays. The investigated method has the following advantages. First, the fabrication processes are easy to duplicate; second, the templates can be simply manufactured; third, the processes make it easy to manufacture ZnO nanoflowers with large areas. At first, a commercially available sapphire with a concave pattern was used as the substrate. After the ZnO seed layer was spun onto the substrate and annealed, an optical glue was layered onto the ZnO seed layer as the carrier. Next, a lift-off technology was used to transfer the ZnO seed layer on the Si wafer and the protrusion patterned ZnO/AB glue seed layer with a matrix structure was obtained. The ZnO/AB glue seed layer had the protrusion and matrix structure; therefore, we could easily synthesize the ZnO nanoflower arrays. We also investigated the effects that different synthesis times and thicknesses of Al sacrificial layers had on the physical and optical properties of the ZnO nanoflower arrays. The following properties of the ZnO nanoflower arrays were investigated: the diameter (D), height (H), density or number of nanorods per unit area (μm^−2^), total surface area per unit area (S, nm^2^), total volume per unit area (V, nm^3^), and photoluminescence (PL) spectrum. Changes in the characteristic emissions in the PL spectra of the ZnO nanoflower arrays under different synthesis times were also investigated.

## 2. Materials and Methods

**Figure 1:**
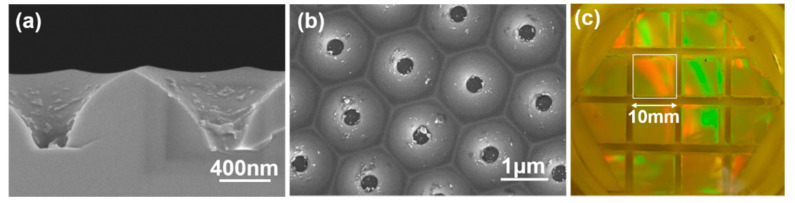
(**a**) Cross-sectional and (**b**) top views of the morphologies of a two-inch sapphire substrate. (**c**) The Al-coated sapphire substrate with heat-resistant tape.

In this study, a lift-off technique was used to transfer a ZnO seed layer with periodic arrays and a protrusion patterned structure to a P-type Si <100> wafer. The hydrothermal method was then used to synthesize ZnO nanorods on the ZnO seed layer to construct ZnO nanoflower arrays. In order to synthesize the ZnO nanoflower arrays, we used a sapphire substrate as a template, which had a patterned concave structure and was provided by Shun Haw Technology Ltd. (Taichung, Taiwan), to prepare the ZnO seed layer with a patterned convex structure [4]. The cross-sectional and top views of the 2 inch sapphire substrate are shown in Figure 1a,b. The average bottom width and height of the protruding nano-structures in sapphire were 0.37 μm and 0.48 μm, respectively.

First, a thermal evaporation coating machine (Chuang Pao Special Precision Industry Co. Ltd., Tainan, Taiwan) was used to deposit Al sacrificial layers of different thicknesses on the sapphire substrate [3]. The deposition process was started at a pressure of 2 × 10^−6^ torr and no gas was introduced during the deposition process. Sample A, which was abbreviated as SC-A, had an Al thickness of 120 nm; sample B (SC-B) and sample C (SC-C) had Al thicknesses of 420 and 720 nm, respectively. Heat-resistant tape (260 °C type, Jaan Yahn Printing Enterprise Co. Ltd., Taichung, Taiwan) was used as an adhesive on the Al-coated sapphire substrate to create an access for sacrificial removal of the Al film from underneath the ZnO seed layer. The area that was used to prepare the ZnO seed layer was 10 mm × 10 mm, as shown in Figure 1c. The spin coating method was used to fully cover the Al-coated sapphire substrate after the ZnO gel had been dipped onto the surface. The ZnO-gel-coated template was then baked at 300 °C for 10 min. The ZnO gel dipping and baking processes were repeated six times to obtain the appropriate thickness. Optical glue (OE-6370HF AB, Sil-More Industrial Ltd., Hsinchu, Taiwan) was used as the carrier of the ZnO seed layer. The Al sacrificial layer was then removed using etching technology; an etching solution of K_3_Fe(CN)_6_:KOH:H_2_O (10 g:1 g:100 mL) was used with an etching speed of the process that varied at 10 min/s. After the Al film was etched, the protrusion patterned ZnO/AB glue seed layers were obtained; then they were lifted off the sapphire substrate and transferred to the P-type Si <100> wafer. The OE-6370HF AB glue was used to attach the ZnO/AB glue seed layers to the Si <100> wafer. Finally, the hydrothermal method was used to synthesize the ZnO nanoflower arrays, because the sapphire substrate had the structure of concave pattern arrays, as Figure 1a,b shows. Therefore, we could use it to prepare a protrusion patterned ZnO seed layer to synthesize ZnO nanoflower arrays.

During the synthesis process, the concentrations of C_6_H_12_N_4_ and Zn(CH_3_COO)_2_ were maintained at 0.02 M, while the duration of the process was increased from 10 min to 60 min. After the ZnO nanoflower arrays were synthesized on the protrusion patterned ZnO seed layer, their X-ray diffraction spectra (XRD, Panalytical, 18 KW rotating anode X-ray generator) were measured to analyze the crystalline phases. Field emission scanning electron microscopy (FESEM, JEOL JSM-6700F, Tokyo, Japan) was used to observe the surface morphologies of the arrays. The prepared protrusion patterned ZnO seed layer and the ZnO nanoflower arrays were cut using a focused ion beam (FIB, FEI Helios 1200+, Hillsboro, OR, USA) system so that the cross-sectional morphologies could be observed. FESEM equipped with energy-dispersive X-ray spectroscopy (EDS, Bruker Quantax 200, Billerica, MA, USA) was used to analyze the undefined residual particles on the surface of the prepared protrusion patterned ZnO seed layer. An iHR550 fluorescence spectrophotometer (Horiba Jobin Yvon, Bensheim, Germany) with a single laser at a wavelength of 325 nm was used as the excitation light source. The PL properties of the ZnO nanoflower arrays in the wavelength range of 350~650 nm at room temperature were measured.

## 3. Results and Discussion

### 3.1. The ZnO Seed Layer Analysis

#### 3.1.1. XRD Analyses

**Figure 2:**
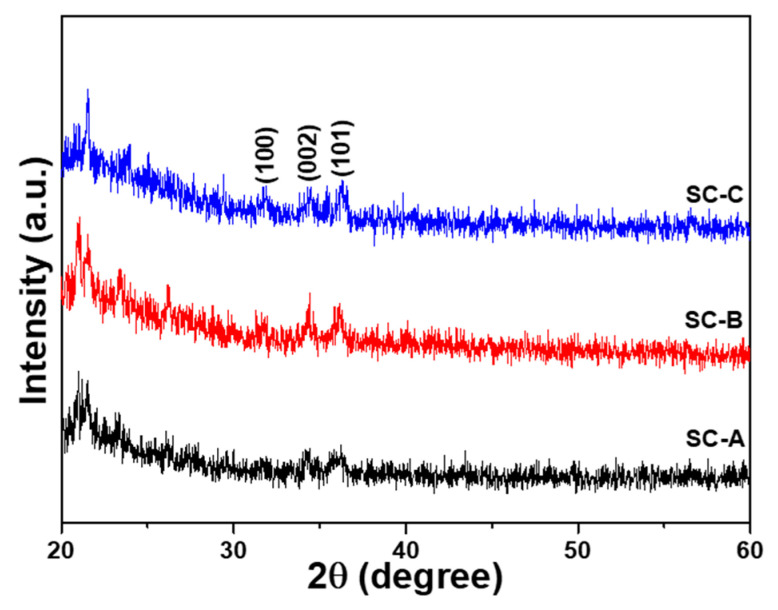
XRD spectra of the ZnO seed layers on substrates with different thicknesses of Al sacrificial layers. Al thicknesses for SC-A, SC-B, and SC-C were 120, 420, and 720 nm, respectively.

The XRD spectra were used to analyze the crystal characteristics of the ZnO seed layers. The XRD spectra for the SC-A, SC-B, and SC-C substrates are shown in Figure 2. There were there unapparent diffraction peaks in all XRD spectra at about 31.72°, 34.40°, and 36.18°, which correspond to the diffraction peaks at the (100), (002), and (101) planes. When the diffraction peaks were compared with the Joint Committee on Powder Diffraction Standards (JCPDS) card number 36-1451, the spectra indicated that the ZnO seed layers have a wurtzite structure with a hexagonal close-packed (HCP) array. For all the XRD spectra shown in Figure 2, the diffraction peak at the (002) plane did not have the highest diffraction intensity, which suggests that the prepared ZnO seed layers did not exhibit the preferred orientation along (002) (the c-axis preferred orientation property).

#### 3.1.2. FE-SEM Analyses

**Figure 3:**
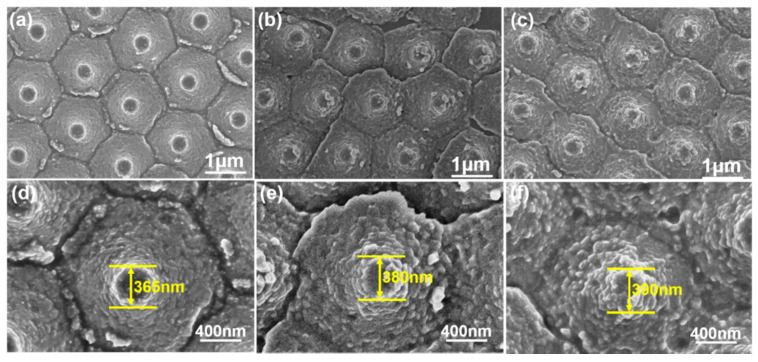
Low-magnification SEM images of the surface of a prepared ZnO seed layer on Al sacrificial layers of different thicknesses: (**a**) 120, (**b**) 420, and (**c**) 720 nm. High-magnification SEM images of the surface of a prepared ZnO seed layer on Al sacrificial layers of different thicknesses: (**d**) 120, (**e**), 420, and (**f**) 720 nm.

Figure 3 shows the FESEM surface images of the prepared ZnO seed layer for Al sacrificial layers of various thicknesses. These images prove that the lift-off technology successfully transferred the ZnO seed layer from the sapphire substrate to the P-type Si <100> wafers to form a protrusion patterned ZnO seed layer. Figure 3a–c shows the low-magnification SEM images of the surface of the prepared ZnO seed layer on Al sacrificial layers of different thicknesses. The images show breakages between the different protrusions; in addition, white particles can be seen in the gaps and on the surfaces of the protrusions. One possible reason for these observations is that the substrate was immersed for a long time in the etching solution, which contained KOH. The KOH reacted with the Al to form white particles of aluminum hydroxide (Al(OH)_3_), which deposited on the surfaces of the protrusions and in the gaps. When EDS was used to analyze the white particles, the Al element was indeed readily detected.

Figure 3d–f shows the high-magnification SEM images of the surface of the prepared ZnO seed layer on Al sacrificial layers of various thicknesses. The particle sizes of the ZnO seed layer for the SC-A, SC-B, and SC-C substrates were calculated using Debye–Scherrer’s equation (Equation (1) below), and they were about 35, 65, and 80 nm, respectively, where, in our study, λ was 1.54 Å, D was the crystalline size, ß was the full width at half maximum (FWHM) values of the (002) plane of the ZnO seed layers, and θ was the diffraction angle:

(1) $$D=\frac{0.9\lambda }{\beta \cos\theta }$$

These images show that the diameters of the top part of the ZnO seed layer were about 365, 380, and 390 nm, respectively. Apparently, as the thickness of the Al sacrificial layer etching time increased, the etching took longer, which increased the particle sizes and the diameters of the ZnO seed layer. These observations could be related to the presence of the Al element. Zhang and Que found that the crystal grain size of Al-doped ZnO changed with the concentration of Al^3+^ ions or Al_2_O_3_; when the concentration of Al increased from 1 at% to 2 at%, the crystal grain size of Al-doped ZnO also increased [21]. In the present study, the concentration of the residual Al in the ZnO seed layer increased with the thickness of the Al sacrificial layer. This observation proves that the amount of an Al-based impurity, such as Al(OH)_3_, that is deposited on the surface of a ZnO seed layer increases with the thickness of the Al sacrificial layer. Therefore, we believe that, as the etching time increases with the thickness of the Al sacrificial layer, the amount of the Al-based impurity also increases, which causes an increase in the particle sizes.

**Figure 4:**
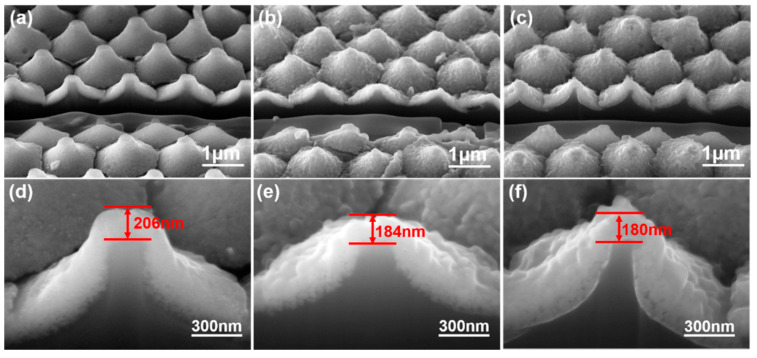
Low-magnification cross-sectional SEM images of the ZnO seed layer on Al sacrificial layers of different thicknesses: (**a**) 120, (**b**), 420, and (**c**) 720 nm. High-magnification cross-sectional SEM images of the ZnO seed layer on Al sacrificial layers of different thicknesses: (**d**) 120, (**e**) 420, and (**f**) 720 nm.

Figure 4a–c shows the low magnification cross-sectional SEM images of the prepared ZnO seed layer on Al sacrificial layers of different thicknesses. The figure shows that the protrusion patterned ZnO seed layer had a matrix structure. Figure 4d–f shows high-magnification cross-sectional SEM images of the prepared ZnO seed layer on Al sacrificial layers of different thicknesses. The thicknesses of the ZnO seed layers on top of the protrusions were measured using the FIB system and determined to be 206, 184, and 180 nm for the SC-A, SC-B, and SC-C substrates, respectively. Figure 4 also shows an important result, which is that even the KOH solution could dissolve or etch the ZnO seed layers the etching effect can thus be neglected, because the thicknesses of all the ZnO seed layers were larger than 180 nm.

**Figure 5:**
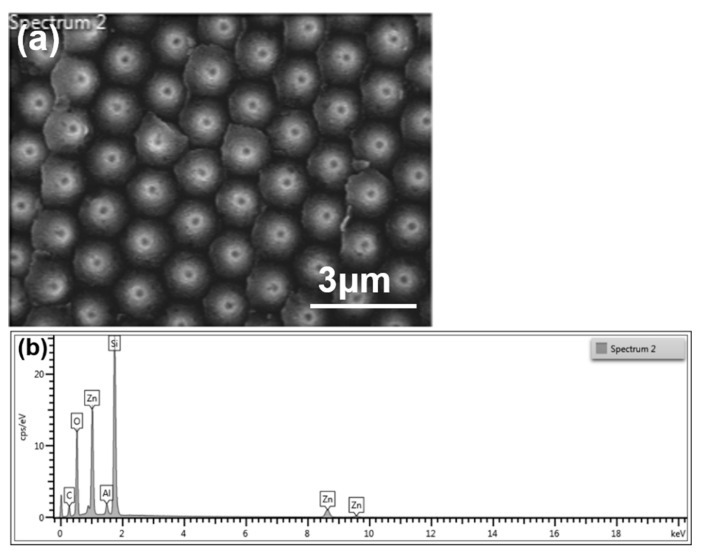
(**a**) Sample used in the EDS analyses; (**b**) the EDS analysis results for the SC-B substrate.

To prove that Al element was residual in the ZnO seed layer, EDS analyses were performed on the surfaces of the ZnO seed layer. Figure 5a shows the result of a SC-B substrate used in the analysis. As can be seen in Figure 5a, the breakages between the seed points are filled with a white material. Figure 5b shows the results of the analysis of the SC-B substrate. As Figure 5b shows, in addition to oxygen (O), zinc (Zn), carbon (C), silicon (Si), and aluminum (Al) elements were detected in all ZnO seed layers synthesized on the SC-B substrate. The results of the EDS analyses of the SC-A and SC-C substrates were similar. The detected signals of Si and C were caused by the silicon wafer and the OE-6370HF AB optical glue. However, we believe that the detected signal of Al was caused by a residual Al-based impurity, such as Al(OH)_3_, which formed during the etching process.

Table 1 compares the EDS results for the SC-A, SC-B, and SC-C substrates. According to the table, as the thickness of the Al sacrificial layer increased from 120 to 720 nm, the percentage of the Al element increased from 0.97% to 3.20%. These results prove that a residual Al-based impurity filled the breakages in the surfaces of the ZnO seed points. The results also indicate that, as the thickness of the Al sacrificial layer increased, there was a greater amount of residual Al-based impurity. Thus, an increase in the amount of the residual Al-based impurity caused an increase in the particle sizes of the ZnO seed layer, as shown in Figure 4d–f.

### 3.2. The ZnO Nanoflower Arrays Analyses

#### 3.2.1. FESEM Analyses

**Figure 6:**
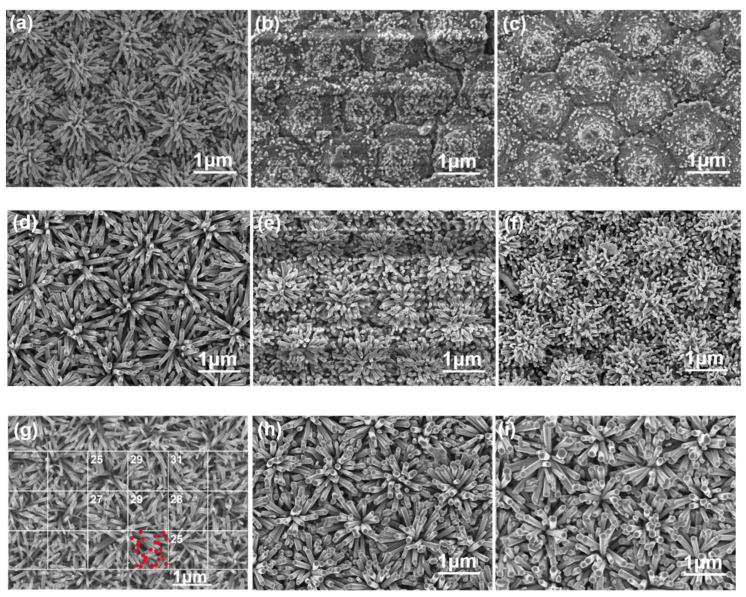
SEM images of the surface of the ZnO nanoflower arrays with a synthesis time of 10 min on Al sacrificial layers of different thicknesses: (**a**) 120, (**b**) 420, and (**c**) 720 nm. With a synthesis time of 30 min on Al sacrificial layers of different thicknesses: (**d**) 120, (**e**) 420, and (**f**) 720 nm. With a synthesis time of 60 min on Al sacrificial layers of different thicknesses: (**g**) 120, (**h**) 420, and (**i**) 720 nm.

Figure 6 shows SEM images of the surface of the ZnO nanoflower arrays on Al sacrificial layers of different thicknesses with synthesis times of 10 min, 30 min, and 60 min, respectively. Compared with the images shown in Figure 3 and Figure 5, these images show that the breakages between the seed points had been patched. This patching effect increased with the synthesis times of the ZnO nanoflower arrays. When the synthesis time was less than or equal to 30 min, incomplete ZnO nanorods were observed on the surfaces of the ZnO nanoflower arrays synthesized on the SC-B and SC-C substrates. The diameters and heights of the ZnO nanorods increased with time, and, as the synthesis times were 10 min and 30 min, the numbers of ZnO nanorods grown decreased with the thickness of the Al sacrificial layer. Figure 6a–c shows that the required synthesis time of the ZnO nanorods increased with the thickness of the Al sacrificial layer. For example, when the synthesis time was 10 min, the ZnO nanorods were readily observed on the SC-A substrate but not observed on the SC-B and SC-C substrates; when the synthesis time was 30 min, the diameters and heights of the ZnO nanorods decreased with the thickness of the Al sacrificial layer. These results suggest that the growth rates of the ZnO nanorods that were synthesized on the SC-B and SC-C substrates were significantly reduced. While obtaining results of the FIB-based cross-sections analyses (reported in Section 3.2.3), when the synthesis time was 60 min, the heights of the ZnO nanorods decreased with the thickness of the Al sacrificial layer. From the results provided in Table 1, we can assume that the reduced growth rate was caused by an increase in the residual Al-based impurities on the surfaces of the ZnO seed layers.

Figure 6 shows that the ZnO nanorods did not only grow perpendicular to the substrates; they also grew along the surface direction of the ZnO seed layer, which was in the radial direction. ZnO nanorods grew with a flower-like structure where each petal had a single rod shape. Therefore, in this study, the ZnO nanorods that were synthesized on a patterned ZnO seed layer grew like a flower as ZnO nanoflower arrays. As shown in Figure 6, the synthesized ZnO nanorods exhibited a uniform morphology (i.e., uniform diameter and height) when the synthesis time was longer than 10 min for the SC-A substrate and longer than 30 min for the SC-B and SC-C substrates. We attribute this uniform morphology to a high growth selectivity and strong polarization along the protrusion ZnO seed layer that allowed the synthesized ZnO nanorods to form a ZnO nanoflower. As Figure 3 and Figure 4 show, the prepared ZnO seed layer had the structure of a hexagonal array with a bump in the center of each hexagon. Because the synthesized ZnO nanorods grew along the vertical substrate, they readily formed the ZnO nanoflower arrays. The use of the patterned sapphire template enabled the ZnO seed layer arrays to easily form matrix structures. Therefore, the investigated technology played a crucial role in synthesizing ZnO nanorods in a radial direction on a patterned ZnO seed layer to form ZnO nanoflower arrays.

#### 3.2.2. XRD Analyses

**Figure 7:**
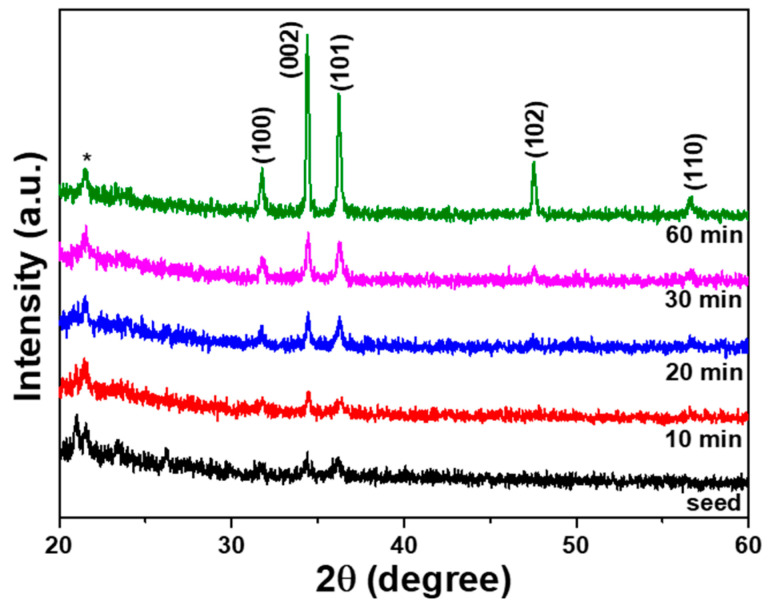
XRD spectra of the ZnO seed layer and ZnO nanoflower arrays on the SC-B substrate as a function of synthesis time; *—AB glue.

Various conditions were changed in order to investigate the effect of the synthesis time on the crystalline and optical properties of the synthesized ZnO nanoflower arrays. First, the synthesis time was changed from 10 min to 60 min, the synthesis temperature was set at 90 °C, and 0.2 M Zn(CH_3_COO)_2_ and 0.2 M C_6_H_12_N_4_ solutions were used. The XRD spectra of the ZnO nanoflower arrays that were synthesized on the SC-B substrate are shown in Figure 7 as a function of synthesis time. The X-ray spectra for the ZnO nanoflower arrays that were synthesized on the SC-A and SC-C substrates were identical to the results shown in Figure 7. The diffraction peaks at the (100), (002), (101), (102), and (110) planes, which corresponded to the peaks located at 2θ values 31.78°~31.80°, 31.38°~34.40°, 36.18°~36.22°, 47.48°~47.52°, and 56.54°~56.58° for different synthesis times, could be readily observed when the synthesis time was equal to or greater than 20 min. When the diffraction peaks are compared with the JCPDS card number 36-1451, the results indicate that the synthesized ZnO nanorods in the ZnO nanoflower arrays had a polycrystalline growth and a wurtzite structure with an HCP array.

**Figure 8:**
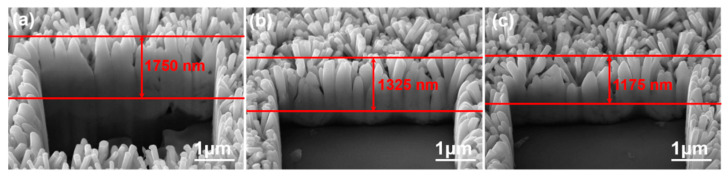
Cross-sectional SEM images of the ZnO nanoflower arrays on Al sacrificial layers of different thicknesses: (**a**) 120, (**b**) 420, and (**c**) 720 nm.

The diffraction intensities of the ZnO nanoflower arrays in five different planes increased with synthesis time. Thus, the crystal properties of the ZnO nanoflower arrays were enhanced with synthesis time. The ZnO nanoflower arrays did not show the characteristic c-axis preferred orientation, because the ZnO nanoflower arrays mainly grew in the direction perpendicular to the ZnO seed layer and spread out like a flower, as shown in Figure 6, Figure 7 and Figure 8. As can be seen in Figure 6b,c, for a synthesis time of 10 min the ZnO nanorods did not completely cover the ZnO seed layer. Therefore, the diffraction intensity of the XRD spectra for the ZnO nanoflower arrays was reduced. The crystalline sizes with the variations in the growth time of the ZnO nanoflower arrays were also calculated using Debye–Scherrer’s equation. The crystalline sizes of the ZnO nanoflower arrays on the SC-B substrate were 11.2, 8.85, 10.5, and 9.33 nm when the synthesis times were 10, 20, 40, and 60 min, respectively.

#### 3.2.3. FIB-Based Cross-Sections Analyses

Figure 8 shows cross-sectional SEM images of the ZnO nanoflower arrays. These images were used to measure the heights and diameters of the ZnO nanoflower arrays on different substrates with a synthesis time of 60 min. After first deducting the thicknesses of the ZnO seed layers for the SC-A, SC-B, and SC-C substrates, which were 206, 184, and 180 nm, the average heights of the ZnO nanoflower arrays were determined to be 1500 nm (the heights were in the range of 1405~1580 nm), 1125 nm (1045~1190 nm), and 975 nm (920~1035 nm), respectively, and the average diameters were 90 nm (the diameters were in the range of 81~98 nm), 103 nm (95~110 nm), and 115 nm (108~123 nm), respectively. In this study, we noticed that the particle sizes of the ZnO seed layer increased with the thickness of the Al sacrificial layer, which caused the heights to decrease and the diameters to increase. ZnO seed layers that had large particle sizes had larger areas for the Zn(CH_3_COO)_2_ and C_6_H_12_N_4_ solutions to deposit ZnO on; thus, more ZnO nanorods could be formed at the same time. In addition, when ZnO nanorods were synthesized on large seed particles, they had a greater chance to merge with each other, which increased the growth rates in the vertical direction (i.e., perpendicular to the ZnO seed layer) and in the radial direction. Therefore, the diameter increased, and the height decreased, with the thickness of the Al sacrificial layer.

Correspondingly, the calculated aspect ratios (H/D) of the ZnO nanoflower arrays decreased from 16.77, to 10.9, and to 8.48 when the SC-A, SC-B, and SC-C substrates were used. Thus, the aspect ratio of the ZnO nanoflower arrays was influenced by the substrates, which affected the relative synthesis rates in the vertical and radial directions. A comparison with Figure 6 shows that, as the same synthesis time was used, the heights of the ZnO nanoflower arrays decreased with the thickness of the Al sacrificial layer. Zhang and Que reported that, as the Al concentration increased, the height of the ZnO nanorods first increased, and then reached a maximum when the Al concentration was 1 at%. When the Al concentration was greater than 1 at%, the growth of the ZnO nanorods was inhibited and, thus, the height of the synthesized ZnO nanorods was reduced [21]. One reason for the decrease in the height of the ZnO nanoflower arrays is that the residual Al-based impurity increased with the thickness of the Al sacrificial layer (Table 1).

To estimate the density (i.e., quantity) of the ZnO nanoflower arrays in a unit area of 1 μm^2^, the image of the ZnO nanoflower arrays was segmented into squares, each with an area of 1 μm^2^, as shown in Figure 6g. At least eight squares were randomly chosen, and the density of the ZnO nanoflower arrays in all selected squares was estimated; the average density was determined from the estimated values. When the thickness of the substrate changed, the residual concentration of the Al-based impurity also changed, which affected the rate of the synthesis of the ZnO nanoflower arrays. Therefore, it was possible to determine how the thickness of the Al sacrificial layer affected the diameter, height, and aspect ratio of the ZnO nanoflower arrays. The average densities of the ZnO nanorods in the ZnO nanoflower arrays that were synthesized on the SC-A, SC-B, and SC-C substrates were 28 μm^−2^, 20 μm^−2^, and 18 μm^−2^, respectively (Table 2). The estimated average diameters, average heights, and average densities of the ZnO nanorods in the ZnO nanoflower arrays were used to calculate the total volume (V) in nm^3^, the total surface area (S) in nm^2^, and the S/V ratio. The results are given in Table 2 for the different substrates with a synthesis time of 60 min.

#### 3.2.4. Photoluminescence of the ZnO Nanoflower Arrays

**Figure 9:**
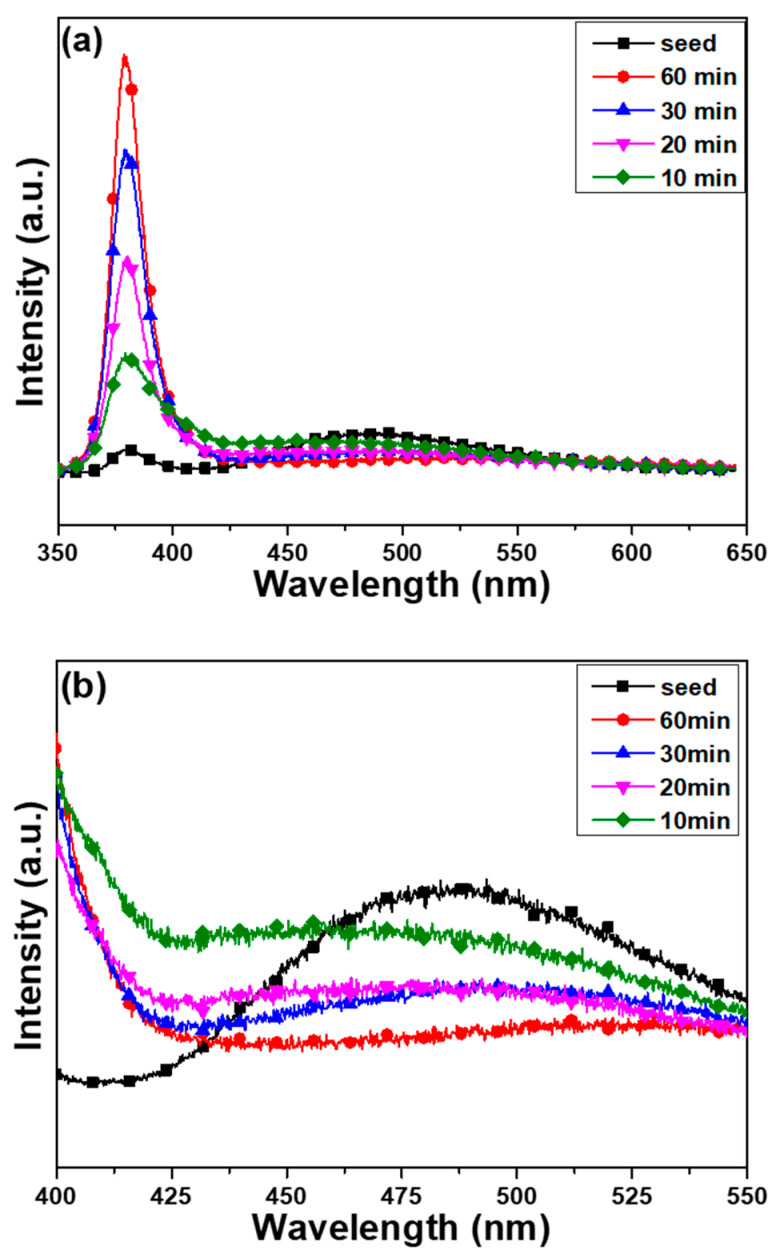
The PL spectra of the ZnO seed layer and ZnO nanoflower arrays on the SC-B substrate as a function of synthesis time: (**a**) in the wide range of 350–650 nm and (**b**) in the narrow range of 400–550 nm.

Figure 9 shows the PL spectra of the ZnO seed layer and the ZnO nanoflower arrays that were synthesized on the SC-B substrate as a function of the synthesis time. As Figure 9 displays, all the PL spectra of the ZnO nanoflower arrays were comprised of a high-intensity emission band, which was centered at 381.0, 380.2, 379.6, and 379.2 nm (UV light, I_UV_). This band was mainly caused by the near-band-edge electron transition between the valence band and the conduction band [22], and a low-intensity broad emission band from 420 to 600 nm (green light, I_G_). The emission intensities of the ZnO nanoflower arrays at the broad emission band of 420~600 nm were lower than those of the ZnO seed layer. The full width at half maximum (FWHM) values for the spectra shown in Figure 9 for ZnO nanoflower arrays with synthesis times of 10, 20, 30, and 60 min were 22.2, 18.4, 17.8, and 16.3 nm, respectively. Lin et al. found that the sharp UV light emission peak at 384 nm could be decomposed into three emission peaks, which were centered at 384, 402, and 424 nm. The peaks centered at 402 and 424 nm were the two donor states (*E_d_*_1_ and *E_d_*_2_), which indicate that the hollow ZnO nanoflower arrays were n-type semiconductors, and the electron transitions from the *E_d_*_1_ and *E_d_*_2_ to the valence band were related to the energies 3.08 and 2.92 eV, and they would release blue light emissions [23]. We believe that the reason for the decrease in the FWHM values at the I_UV_ peaks was the decrease of the intensities in the two donor states.

The broad visible emission band corresponds to the transitions between the near-band-edge and the local levels, which are caused by intrinsic defects that exist in the synthesized ZnO nanomaterials [24], or corresponds to the electron transitions between the valence band and the deep levels, where the deep levels are ascribed to the oxygen vacancies, structural defects, Zn residues, and impurities [23,25]. Examples of intrinsic defects include zinc vacancy (V_Zn_; bandgap energy (*E*g) = 3.06 eV; and emission light ~405 nm), interstitial zinc (Zn_i_; *E*g = 2.90 eV; and emission light ~428 nm), antisite defect (O_Zn_; *E*g = 2.38 eV; and emission light ~521 nm), interstitial oxygen (O_i_; *E*g = 2.28 eV; and emission light ~544 nm), and oxygen vacancy (V_O_; *E*g = 1.62 eV; and emission light ~765 nm) [24]. Therefore, the emission of I_G_ is believed to be caused by combinations of different defects at local levels, including an interstitial oxygen defect (~544 nm) and an antisite defect (~521 nm) in the ZnO seed layer and ZnO nanoflower arrays.

Figure 9a shows that the emission intensity of I_UV_ increased, while the emission intensity of I_G_ decreased with the synthesis time of the ZnO nanoflower arrays. In addition, the I_UV_ values of the ZnO nanoflower arrays were greater than those of the ZnO seed layer; correspondingly, the I_G_ values of the ZnO nanoflower arrays were smaller than those of the ZnO seed layer. When the synthesis time increased from 10 min to 60 min, the intensity of I_G_ decreased from 170 to 83 (a.u.), and the intensity of I_UV_ increased from 594 to 2068; the I_G_/I_UV_ ratio decreased from 0.285 to 0.040. Table 3 compares the I_UV_ values, I_G_ values, and I_G_/I_UV_ ratios of the ZnO nanoflower arrays with different substrates and synthesis times. When the images of the ZnO nanoflower arrays shown in Figure 6 are compared with the variations in the PL spectra shown in Figure 9a, the results prove that the synthesis time is an important factor that affects the PL properties of the synthesized ZnO nanoflower arrays. It is noted that the PL properties of the ZnO nanoflower arrays that were synthesized on the SC-A and SC-C substrates showed the same trends as the ZnO nanoflower arrays that were synthesized on the SC-B substrate.

It is well known that the crystal quality of ZnO nanoflower arrays is closely associated with the intensity of the near-band-edge emission [26]. Both the antisite defect and the interstitial oxygen defect have close relativities with the emission intensity in the range of 420~575 nm [26]. As Figure 9 shows, the emission intensities of the ZnO nanoflower arrays at the broad emission band of 420~575 nm were lower than that of the ZnO seed layer; however, the emission intensities of the ZnO nanoflower arrays at the strong emission peak of ~381 nm were higher than the emission intensities of the ZnO seed layer. These results suggest that different defects existed in the prepared ZnO seed layer and contributed to its poor crystal quality, as shown in Figure 2 and Figure 7. Therefore, the PL property of the ZnO nanoflower arrays depended on the synthesis time, considering the fact that their crystal quality improved to a certain extent with the synthesis time.

The greater the amount of antisite defects and interstitial oxygen defects that existed in the ZnO seed layer and ZnO nanoflower arrays, the stronger the emission intensity of I_G_ in the broad emission band of 420~575 nm was. In general, the maximum emission intensity of I_G_ can be used to judge the crystal quality of ZnO nanomaterials, because it indicates defects. Figure 9b shows that the zinc vacancy defect, which emits light at ~405 nm, decreased with the synthesis time, although the interstitial zinc defect, which emits light at ~428 nm, was not observed in the ZnO seed layer or ZnO nanoflower arrays. The figure also shows that the emission peak of the broad emission band for the ZnO seed layer was located at 486 nm and the emission peak of the broad emission band for the ZnO nanoflower arrays shifted from 462 to 537 nm as the synthesis time increased from 10 min to 60 min. These results indicate that when the ZnO nanoflower arrays were synthesized on a patterned ZnO seed layer, the antisite defects and the interstitial zinc defects could be repaired during the synthesis of the ZnO nanoflower arrays. However, the repair rate of the antisite defects was lower than that of the interstitial zinc defects, as the wavelength for the maximum emission intensity shifted to a higher value. The decreases in the two defects over time prove that the crystal qualities of ZnO nanoflower arrays were enhanced with the synthesis time. Thus, ZnO nanoflower arrays with a high emission intensity of I_UV_ were successfully obtained when the synthesis time was extended.

**Figure 10:**
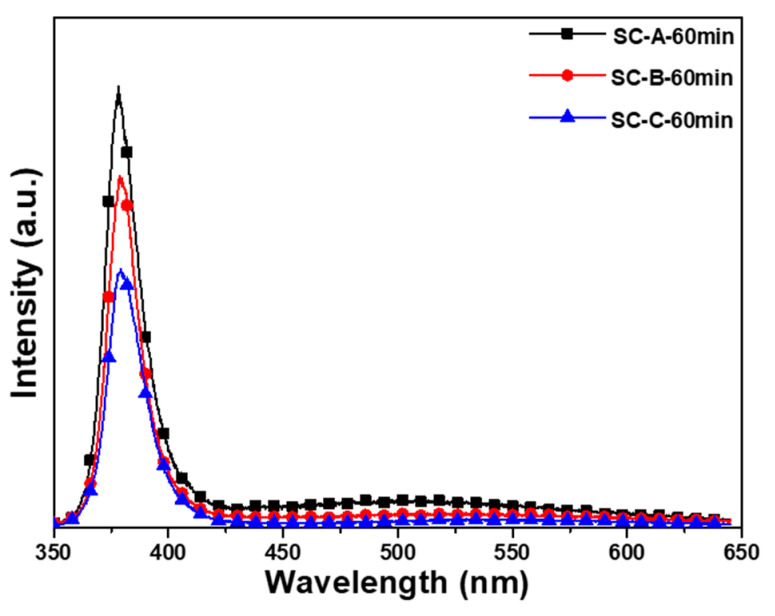
The PL spectra of the ZnO nanoflower arrays with a synthesis time of 60 min on different substrates.

**Figure 11:**
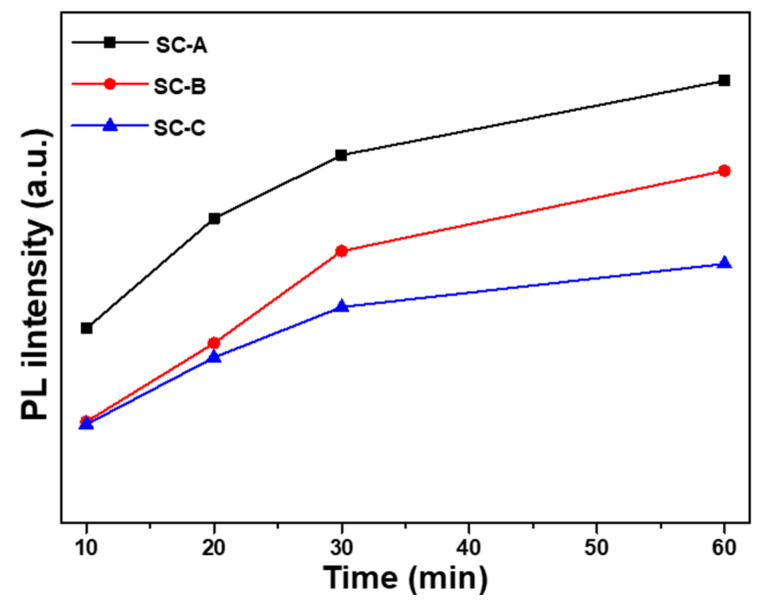
Emission intensities of I_UV_ of the ZnO nanoflower arrays using different synthesis times and substrates.

The results of this study prove that when different synthesis times were used, there were fewer defects, shown as I_G_, in the ZnO nanoflower arrays than in the ZnO seed layer; the emission intensities of I_UV_ from the ZnO nanoflower arrays were also weaker than those from the ZnO seed layer. Figure 10 shows a comparison of the PL spectra of the ZnO nanoflower arrays with a synthesis time of 60 min on different substrates. The emission intensities of both I_UV_ and I_G_ increased with synthesis time. Table 2 shows that when the synthesis time was 60 min, the diameter, height, aspect ratio, density, total surface area, and total volume decreased with the thickness of the Al sacrificial layer. The contacting surface size of the ZnO nanoflower arrays determined the volume that received the excitation laser light and the volume that emitted the PL spectra. Therefore, the total surface area was the most important factor that affects the emission intensity. When Figure 6 and the results in Table 2 are compared, we observe that the total surface area of the ZnO nanoflower arrays decreased with the thickness of the Al sacrificial layer. Figure 11 shows the emission intensities of I_UV_ of the ZnO nanoflower arrays as a function of the synthesis time and thickness of the Al sacrificial layer. This figure shows that, for the same synthesis time, the emission intensity of I_UV_ decreased with a decrease in thickness of the Al sacrificial layer. These results match the results shown in Figure 6. Thus, a comparison of the variations of the PL spectra shown in Figure 10 and Figure 11, combined with the results in Table 3, proves that the Al sacrificial layer was another important factor that affected the PL properties of the ZnO nanoflower arrays.

**Figure 12:**
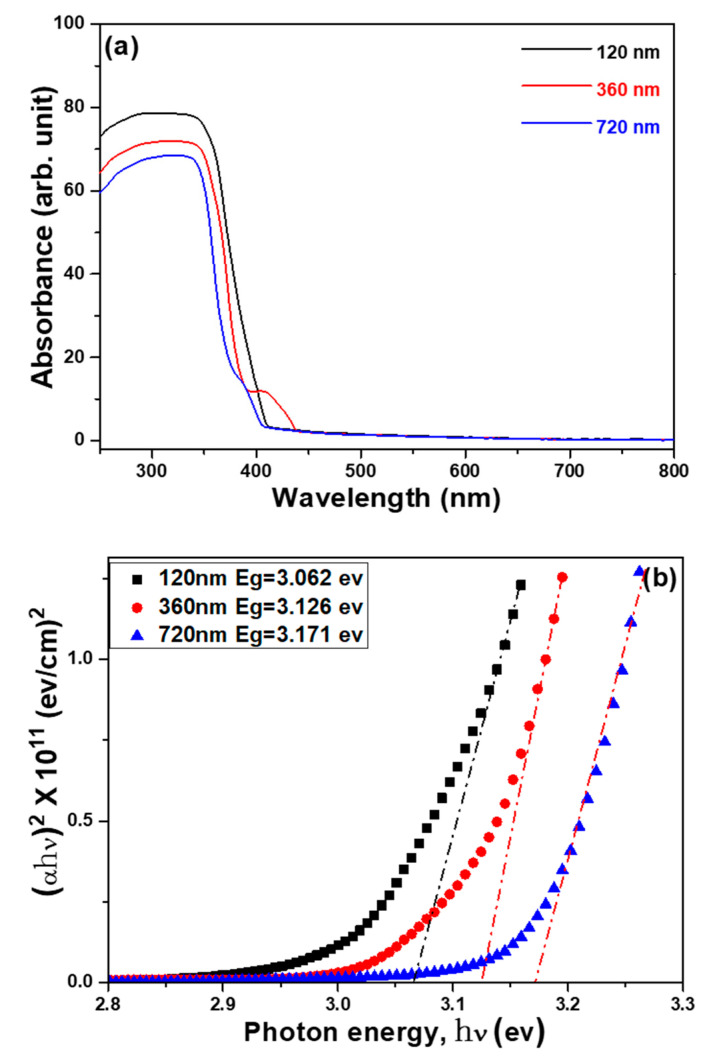
(**a**) UV-visible absorption spectra and (**b**) Tauc plots from UV-visible absorption spectroscopy of the ZnO nanoflower arrays using different substrates.

The drawings of the UV-visible absorption spectroscopy of the ZnO nanoflower arrays for samples with the synthesis time of 60 min and Al sacrificial layers of 120, 240, and 720 nm are shown in Figure 12a, and their Tauc plots corresponding to each UV-visible spectrum are shown in Figure 12b. UV-visible absorption spectra of the ZnO nanoflower arrays revealed the optical absorption in the UV region, which could be recognized by the migration of electrons from the valance band to the conduction band [18,27]. UV-visible spectra revealed that their absorption rates decreased with the thickness of the Al sacrificial layer. The calculated band gaps for samples synthesized on the Al sacrificial layer with the thicknesses of 120, 360, and 720 nm were 3.026, 3.126, and 3.171 eV, respectively. To explore the defects’ states and charge carrier dynamics, the results in Figure 12 can be compared with the PL spectra shown in Figure 10. All the PL spectra of the ZnO nanoflower arrays showed one major peak of band edge emission at around 379–381 nm and one broad hump in the visible region 420~600 nm. The decrease of the emission intensity in the visible region suggests a reduction in the defect density states in the ZnO nanoflower arrays.

**Figure 13:**
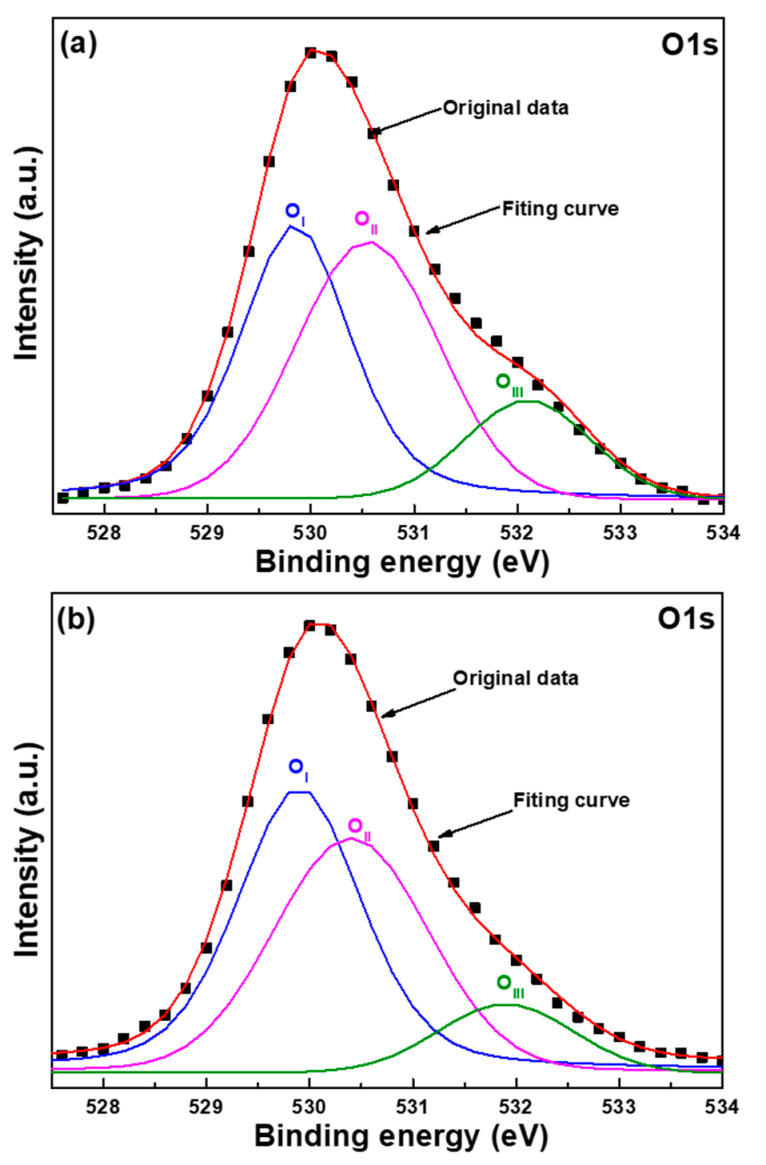
The drawings of O_1s_ peak of the ZnO nanoflower arrays using different substrates and Al sacrificial layers of (**a**) 120 nm and (**b**) 720 nm.

For further discussing the effect of the thickness of the Al sacrificial layer, we used an X-ray photoelectron spectroscopy (XPS) technique to measure the chemical-bonding state of oxygen. These measured results were used to find the relationship between the defects caused by the different substrates and the optical properties of the ZnO nanoflower arrays. The drawings of the O_1s_ peak of the ZnO nanoflower arrays are of samples with a synthesis time of 60 min and Al sacrificial layers of 120 and 720 nm. The typical surface O_1s_ peaks of the ZnO nanoflower arrays were centered at 530.1 ± 0.1 eV and fitted by three Gaussian components of O_I_, O_II_, and O_III_ peaks, as Figure 13a,b shows. The O_1s_ peak was divided into three peaks, O_I_, O_II_, and O_III_, which were centered at 529.8 ± 0.1 eV, 530.3 ± 0.1 eV, and 532.1 ± 0.1 eV, respectively. For the ZnO nanoflower arrays, the bond energy for the component of the O_I_ peak was caused by the Zn^2+^-O^2−^ bond, the bond energy for the component of the O_II_ peak was caused by the oxygen vacancies, and the bond energy for the component of the O_III_ peak was caused by the chemical absorption of oxygen on the surfaces [28].

The areas of the O_1s_ peaks measured according to the XPS spectra of the ZnO nanoflower arrays are compared in Table 4 as a function of the thickness of the Al sacrificial layer. As the thickness of the Al sacrificial layer of the ZnO nanoflower arrays increased from 120 to 720 nm, the area of the O_I_ peak increased from 45.26% to 48.54%, the area of the O_II_ peak decreased from 43.06% to 40.93%, and the area of the O_III_ peak decreased from 11.68% to 10.54%. Apparently, the area of the O_II_ peak of the ZnO nanoflower arrays decreased with the increase in the thickness of the Al sacrificial layer, which proves that the oxygen vacancies of the ZnO nanoflower arrays decrease with the increase in the thickness of the Al sacrificial layer. These results also suggest that the defects in ZnO nanoflower arrays decrease with the increase in the thickness of the Al sacrificial layer. Because the oxygen vacancies decreased with the thickness of the Al sacrificial layer, these results indirectly prove that the intensity of the I_G_ value of ZnO nanoflower arrays decreases with the thickness of the Al sacrificial layer, which matches the measured results shown in Figure 10.

## 4. Conclusions

In this study, ZnO seed layers that had a protrusion patterned array structure were prepared on Al sacrificial layers of different thicknesses. For Al sacrificial layers that were 120, 420, and 720 nm thick, the particle sizes of the ZnO seed layers were about 35, 65, and 80 nm, the diameters of the top parts of the ZnO seed layers were about 365, 380, and 390 nm, and the ZnO seed layers were 206, 184, and 180 nm thick, respectively. The SEM and EDS analyses indicated that when a thick Al sacrificial layer was deposited, a longer etching time was needed and more Al-based impurity was residual. The heights of the ZnO nanoflower arrays decreased and the diameters increased with the thicknesses of the sacrificial Al layer. The results also indicated that the diameter increased and the height, aspect ratio, density, total surface area (S), total volume (V), S/V ratio, I_UV_, I_G_, and I_G_/I_UV_ ratio of the ZnO nanoflower arrays decreased with the thicknesses of the Al sacrificial layer.

## Figures and Tables

**Table 1:** Results of the EDA analyses of prepared ZnO seed layers for the three substrates. wt% and at%: percentage by weight and atomic percentage.

	SC-A		SC-B		SC-C	
Element	wt%	at%	wt%	at%	wt%	at%
O	40.04	72.74	45.32	75.86	43.45	74.40
Zn	58.99	26.22	51.69	21.17	53.35	22.35
Al	0.97	1.04	2.99	2.97	3.20	3.25
Total	100.0	100.0	100.0	100.0	100.0	100.0

**Table 2:** Diameter (D), height (H), aspect ratio (H/D), density, total surface area (S), total volume (V), and S/V ratio of ZnO nanoflower arrays on different substrates with a synthesis time of 60 min.

Substrate	SC-A	SC-B	SC-C
Diameter (nm)	90	103	115
Height (nm)	1500	1150	975
Aspect ratio (H/D)	16.7	11.0	8.12
Density (μm^−2^)	28 ± 3	20 ± 2	18 ± 2
Total surface area (nm^2^)	1.20 × 10^7^	7.76 × 10^6^	6.82 × 10^6^
Total volume (nm^3^)	2.67 × 10^8^	1.99 × 10^8^	1.98 × 10^8^
S/V ratio	4.51 × 10^−2^	3.90 × 10^−2^	3.44 × 10^−2^

**Table 3:** Variations in the I_UV_ and I_G_ values and the I_G_/I_UV_ ratio of the ZnO nanoflower arrays with different synthesis times and substrates.

	SC-A			SC-B			SC-C		
Time (min)	I_UV_	I_G_	I_G_/I_UV_	I_UV_	I_G_	I_G_/I_UV_	I_UV_	I_G_	I_G_/I_UV_
10	1156	337	0.291	594	170	0.285	581	145	0.249
20	1804	263	0.145	1074	119	0.110	971	127	0.130
30	2161	257	0.119	1597	116	0.072	1275	110	0.086
60	2597	163	0.062	2068	83	0.040	1522	49	0.032

**Table 4:** Areas of O_1s_ peaks of ZnO nanoflower arrays as a function of thickness of the Al sacrificial layers.

Thickness of Al	120	360	720
O_I_	45.26%	46.67%	48.54%
O_II_	43.06%	41.96%	40.92%
O_III_	11.68%	11.37%	10.54%

## Data Availability

Not applicable.
